# Engineering Human Myocardium: Integrating the Maturation of hiPSC-Derived Cardiac Myocytes Across Genetic, Structural, Physiological and Multicellular Systems

**DOI:** 10.3390/cells15111019

**Published:** 2026-06-01

**Authors:** Nora Hosny, Houda Cohen, John Bauer, Jeff Schreifels, Rachel Lin, Brian R. Thompson, Joseph M. Metzger

**Affiliations:** 1 Department of Integrative Biology and Physiology, University of Minnesota Medical School, Minneapolis, MN 55455, USA; ahmedn@umn.edu (N.H.); cohen461@umn.edu (H.C.); bauer761@umn.edu (J.B.); schre033@umn.edu (J.S.); lin00940@umn.edu (R.L.); thom1709@umn.edu (B.R.T.); 2 Medical Biochemistry and Molecular Biology Department, Faculty of Medicine, Suez Canal University, Ismailia 41522, Egypt

**Keywords:** hiPSC-CMs, cardiac myocyte maturation, sarcomere organization, cardiac tissue engineering, multiscale maturation cues

## Abstract

The landscape of human cardiac biology was transformed by the discovery that adult somatic cells can be reprogrammed into induced pluripotent stem cells, enabling patient-specific disease modeling, drug testing, and regenerative strategies without the prior ethical or biological constraints. Subsequent advances in directed differentiation made the generation of human iPSC-derived cardiac myocytes reliable and scalable. Despite this progress, a central limitation has remained: these cells are developmentally immature, resembling fetal cardiac myocytes in structure, metabolism, and function. This immaturity restricts their utility for modeling adult-onset disease, predicting drug responses, and achieving clinical translation. Maturation is now understood as a multifactorial symphony, requiring coordinated molecular, structural, and environmental inputs rather than single interventions. As a result, the field is shifting toward integrative approaches that incorporate 3D architecture, multicellular systems, and biomimetic environments to better replicate native cardiac tissue. While fully adult-like myocardium remains an ongoing goal, advances in bioengineering and system-level design are narrowing the gap, with success increasingly defined by the generation of functional cardiac tissue rather than isolated cell maturity.

## 1. Overview

Cardiovascular diseases (CVDs) are the leading cause of global morbidity and mortality [1]. Recent epidemiological projections suggest a 90% increase in global crude CVD prevalence and a 73.4% rise in projected crude mortality between 2025 and 2050, largely driven by population growth and aging; however, age-standardized CVD mortality is projected to remain relatively stable (−3.6%), while age-standardized mortality is expected to decrease by 30.5% [2]. This escalating global burden underscores the critical need for human relevant experimental platforms that can accurately recapitulate cardiac physiology and pathology.

### Human-Based Models of Cardiac Muscle

For many decades, rodent models have served as the dominant experimental platform for cardiac research, largely due to their practical advantages in breeding efficiency, genetic engineering, and accessibility of cardiac tissue [3,4]. They have yielded invaluable mechanistic insights and remain indispensable for many aspects of cardiovascular biology. However, fundamental interspecies differences between rodents and humans place some limits on the full translational relevance of rodent models. A mouse heart is not simply a smaller human heart; rather, it operates under distinct physiological constraints, including markedly higher resting heart rates and different electrical activation, repolarization dynamics and contractile indices [3,5,6].

One striking example lies in the shape and duration of the cardiac action potential (AP). Murine cardiac muscle exhibits a markedly shorter ventricular AP that lacks a clear plateau phase. This difference is readily reflected at the organ level in the electrocardiogram (ECG): the QT interval lasts only ~50–100 ms in mice, versus ~400 ms in humans [5]. While the initial depolarization phase (phase 0) is broadly similar across species, major divergence occurs during repolarization. In human ventricular cardiac myocytes, repolarization is primarily mediated by the rapid and slow components of the delayed rectifier potassium currents, I_Kr_ and I_Ks_, respectively [5]. In contrast, murine ventricular repolarization depends largely on the transient outward potassium current (I_to_), while additional currents such as I_K,slow1_, I_K,slow2_, and I_ss_ also contribute [5]. These differences in voltage-gated potassium channel composition have important functional consequences. For instance, genetic mouse models of human long QT syndromes (LQT1 and LQT2), which are associated with loss of I_Ks_ and I_Kr_ respectively, do not fully reproduce the human phenotype, underscoring the limitations of rodent systems for modeling human arrhythmogenic disorders [5].

Beyond electrophysiology, contractile machinery and signaling also diverge across species. For example, at the sarcomere level adult mouse cardiac myocytes predominantly express α-myosin heavy chain whereas human cardiac myocytes mainly express the β-myosin heavy chain isoform [7]. Phosphodiesterase (PDE) regulation also differs, with PDE4 shaping cAMP signaling linked to L-type Ca^2+^ current in rodents, whereas PDE3 is the dominant regulator of β-adrenergic/cAMP signaling in human cardiac myocytes [8,9].

These disparities underscore the need for human-based models. Human pluripotent stem cell technologies have enabled the generation of patient-specific cardiac myocytes [10]. Capturing disease-associated mutations and human genetic background diversity, that cannot be faithfully reproduced in mouse models [3,11,12,13,14], enables applications in regenerative medicine, drug screening, and disease modeling [8,15,16,17]. Since the landmark discovery of human-induced pluripotent stem cell (hiPSCs) by Takahashi and Yamanaka in 2007, protocols for cardiac differentiation have evolved significantly, enabling the efficient and reproducible production of hiPSC-derived cardiac myocytes (hiPSC-CMs) [18,19,20,21,22,23,24,25].

**Figure 1:**
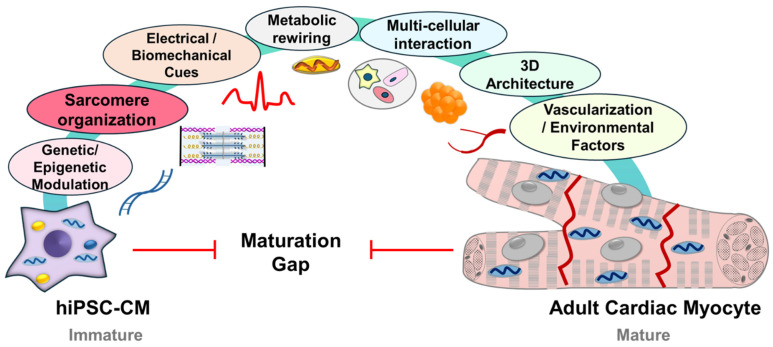
Path to myocardial maturity. A conceptual diagram showing the transition (left to right) from immature, fetal-like hiPSC-CMs to mature, adult-like cardiac myocytes, highlighting key intervention nodes including genetic/epigenetic modulation, sarcomere organization, electric/biomechanical cues, metabolic rewiring, multicellular interaction, 3D architecture, and vascularization/environmental factors.

Despite these advances, hiPSC-CMs remain immature and retain a fetal-like phenotype [26,27]. It is increasingly recognized that hiPSC-CM maturation is not merely a cell-autonomous process but a complex structural and environmental one, requiring a myriad of biophysical and biochemical cues (Figure 1) [28,29]. While two-dimensional (2D) systems provide experimental control, they do not fully recapitulate the anisotropic and complex hemodynamic environment of the adult heart [30,31]. In 2D cultures, hiPSC-CMs often experience maturation arrest, characterized by disorganized myofibrils, altered sarcomere isoform transitions, spontaneous beating, and a reliance on glycolytic metabolism rather than the fatty acid oxidation (FAO) typical of the adult myocardium [29,31,32,33,34].

To address this, the field is shifting toward three-dimensional (3D) models to better mimic the in vivo cardiac microenvironment. As detailed below, 3D systems enable the integration of structural alignment, mechanical loading, and electrical stimulation, promoting more advanced maturation [35,36,37].

## 2. Lessons from Postnatal Heart Maturation

The mammalian heart undergoes extensive postnatal maturation with changes in gene expression, metabolism, structural organization and overall function [38]. Physiological inputs including oxygen tension, hormonal changes, hemodynamic strain, and increased vasculature have all been implicated in the complex process of going from a neonatal heart to an adult heart. Understanding what changes occur through this process will help define maturation benchmarks for iPSC-CMs. Briefly, we summarize here the differences between neonatal and adult cardiac myocytes.

Morphologically, neonatal cardiac myocytes lack the classic rod-shaped morphology of adult cardiac myocyte, with disarrayed sarcomeres, sarcomere lengths of ~1.6 µm [31], and mitochondria that are disorganized [39]. Neonatal myocytes have nascent transverse tubules (T-tubules) which reduces the efficiency of calcium-induced calcium-release (CICR) from the sarcoplasmic reticulum [40]. Adult cardiac myocytes are rod-shaped with aligned sarcomeres and average baseline sarcomere lengths of ~2.1 µm [41]. Here, the mitochondria network expands and mitochondria align with sarcomeres [42].

Metabolically, neonatal myocytes are mainly glycolytic, relying on glucose as their main energy substrate [38]. During development, increases in circulating thyroid hormones and glucocorticoids contribute to increased mitochondrial biogenesis and increased fatty acid metabolism [43,44]. These consortium of metabolic changes results in the myocyte using fatty acid oxidation as the main source of ATP production [38]. This metabolic switch remains in the adult heart, although in heart failure this switch can reverse [45].

Cardiac myocyte maturation also includes increased expression of adult isoforms of ion channels and calcium handling proteins. These changes result in decreased resting membrane potential, increased conduction velocity and increased action potential duration [31,46]. Calcium concentrations increase at rest and at peak amplitude of the calcium transient [47]. Together these changes lead to mature excitation contraction coupling as required for effective adult myocardium performance.

The sarcomere is the functional unit of cardiac contractility [48,49]. Important sarcomeric proteins undergo isoform switching during early postnatal development [50]. These switches result in altered contractile function. β-myosin heavy chain (β-MHC) is the main myosin motor in the adult human heart. During early postnatal development, there is an increase in the α-myosin heavy chain (α-MHC) protein isoform content [51]. This upregulation results in ~5–10% α-MHC at the protein level (up to ~30% at the mRNA level) and the remaining ~90–95% being β-MHC [52,53,54,55,56]. This small but important amount of α-MHC results in increased contractility that is critical to adult heart performance [51,52,56]. This MHC isoform switch is reversible as noted during heart failure and thought to contribute to the poor contractile performance [52].

Another critical isoform switch in the sarcomere is the switch from slow skeletal troponin I (ssTnI (TNNI1)) to cardiac troponin I (cTnI (TNNI3)) [26]. In humans, ssTnI predominates throughout fetal life and is gradually replaced by cTnI, with completion by approximately 9 months postnatally [26,57,58]. In mice this switch starts shortly after birth and is completed by ~P21 [59]. In the adult heart, 100% of TnI is cTnI and this switch is irreversible. Here, cTnI increases lusitropy compared to ssTnI and confers increased lusitropy with β-adrenergic signaling due to phosphorylation of two serine residues in its unique N-terminal domain of cTnI [60].

The hormonal milieu is an important feature of cardiac development in vivo. The transition from fetal to adult cardiac phenotypes is driven by a complex hormonal orchestration involving the somatotropic axis (IGF-1), thyroid hormones (T3), and glucocorticoids, which collectively regulate sarcomere organization, T-tubule development, and electrophysiological refinement [61,62,63,64]. For example, T3 promotes the fetal to adult myosin heavy chain isoform transition (i.e., increases α-MHC percentage), increasing sarcomere length, and shifting metabolism toward higher oxygen consumption rates in hiPSC-CMs [63]. Sex steroid signaling adds a further dimension. HiPSCs possess the molecular machinery, including progesterone receptor (PR) expression, to respond to steroid hormones prior to the onset of directed differentiation, suggesting that steroid signaling can shape developmental trajectories from the onset of reprogramming and differentiation [65]. Steroid receptor activation as an estrogen-related receptor is closely linked to mitochondrial maturation and the developmental metabolic shift toward oxidative phosphorylation and fatty acid utilization, hallmarks of adult cardiac myocyte physiology [66]. Therefore, cardiac platforms without endocrine cues may fail to fully reproduce adult-like and sex specific cardiac phenotypes [67]. Incorporating hormonal signaling into maturation protocols would likely improve the physiological fidelity of iPSC derived cardiac models, with particular importance for studying sex-biased cardiovascular disease, drug responses, and adaptive remodeling.

## 3. Defining Maturation Benchmarks

**Figure 2:**
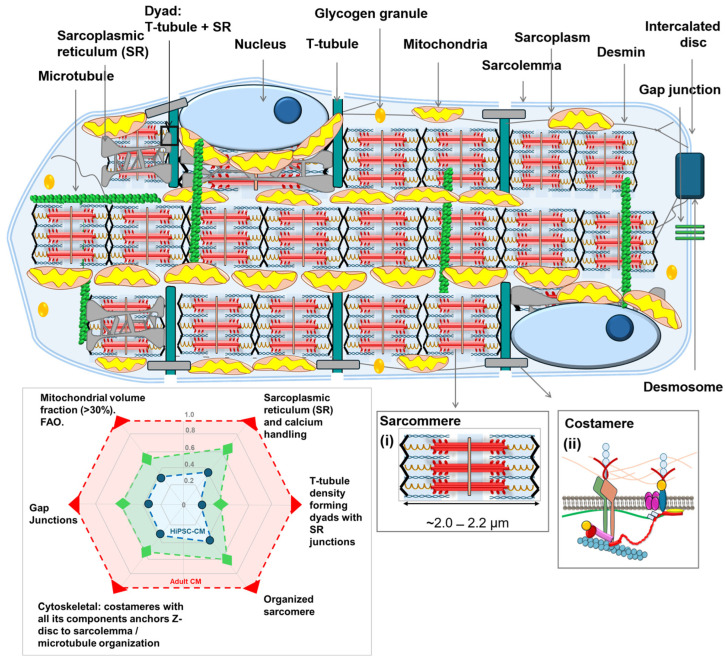
Schematic representation of the intracellular structure of a mature human cardiac myocyte and comparative maturation assessment with hiPSC-CMs. The main panel illustrates a longitudinal section of a human cardiac myocyte highlighting key structural and functional components: sarcomeres (filaments with Z-disks), transverse tubules (T-tubules, teal), sarcoplasmic reticulum (SR, gray), mitochondria (yellow-orange), nucleus (baby blue), gap junctions (green), costameres and associated cytoskeleton (gray rectangle), and contractile actin–myosin filaments (blue and red). Small yellow spheres represent glycogen granules. Insets provide magnified views of: (**i**) the sarcomeric unit with Z-disk, myosin, actin, and titin organization; and (**ii**) a representative costamere complex including integrins, focal adhesion proteins, and dystrophin-associated glycoprotein complex. The lower left panel presents a radar (spider) chart summarizing relative maturation levels of hiPSC-CMs compared to adult human cardiac myocytes across six functional domains: sarcoplasmic reticulum and calcium handling, T-tubules and dyadic structures, mitochondrial composition and fatty acid oxidation (FAO), sarcomere organization, gap junction formation, and cytoskeleton including costamere network integrity and microtubules. The blue polygon represents immature hiPSC-CMs, green shows partially matured cells after experimental enhancement strategies, and red denotes the adult human cardiac myocyte benchmark. Axis values represent qualitative literature-based maturation scores (scale 0–1; 0 = fully immature, 1 = adult human cardiac myocyte reference), all values are relative literature-based estimates. This schematic provides a reference framework for assessing hiPSC-CM maturation and guiding targeted interventions to achieve adult-like cardiac phenotypes.

Maturation of cardiac myocytes involves a myriad of changes as discussed above so no single change on its own will suffice as a benchmark of a mature myocyte. A combination of structural changes, functional changes, genetic program switches and metabolic switches are all needed to attain maturity (Figure 1). As such, comparison to the adult cardiac myocyte for all the “stalled” phenotypes and genotypes would be the best benchmark (Table 1). As noted above, there are many differences between immature and mature cardiac myocytes (Figure 2) and so we highlight a few benchmarks we see as critical.

As the sarcomere is the functional unit of the heart, isoform switches in the sarcomere are critical for a mature myocyte [50]. Recently, it has been shown that α-MHC is absent in iPSC-CM and adenoviral expression of α-MHC leads to positive inotropy [7]. This finding highlights that increasing functional maturity of iPSC-CM arises in part by increases in α-MHC expression. While this MHC switch is important functionally it should be noted that it is reversible. Accordingly, if α-MHC expression is down, it could be from being immature or from being diseased. It is also known that the critical switch from ssTnI to cTnI does not occur in iPSC-CM [59,60]. Using gene editing with inducible regulatory control, the forced expression of cTnI results in faster relaxation at baseline, and faster relaxation with isoproterenol [68]. This finding is evidence that cTnI expression will result in functional changes toward more adult-like function. This switch is irreversible so as a maturation marker it is ideal as it does not revert back in disease [59].

**Table 1:** Multi-dimensional benchmarks of maturation in hiPSC-derived vs. adult cardiac myocytes.

**A. Structural/Morphological**			
**Parameter**	**Adult Human CM**	**hiPSC-CM (Standard 2D)**	**Reference(s)**
Cell shape	Rod-shaped; length:width ~7:1; anisotropic	Round or polygonal; aspect ratio 2–3:1; isotropic	[69,70,71]
Cell size and hiPSC-CM dimensions (in vitro)	~150 µm length, 20 µm width, 15 µm height ~45,000 µm^3^ in volume	Early (20–40 d of differentiation): diameter ~5–10 µm and heights of ~5 µm vs. 30 µm in length, 10 µm in width, height ~6.7 μm and 2000 µm^3^ in volume with prolonged culture (> 40 d) Area: Early stage: ~480 µm^2^; prolonged culture: ~1716 µm^2^ Perimeter (131 µm early vs. 284 µm in prolonged culture), with decrease in the circularity index (0.38 vs. 0.28)	[31,71,72]
Sarcomere length	~2.0–2.2 µm	~1.6–1.8 µm; disorganized Z-disks; no clear M-band	[31,70,71,73,74,75]
T-tubule system	Extensive; L-type Ca^2+^ channel/RyR2 dyads; synchronous CICR	Absent; not formed even with prolonged culture	[31,73,76]
Intercalated disks/Connexin-43 (Cx43)	Mature; Cx43 polarized to longitudinal ends; fast conduction	Diffuse circumferential Cx43; no polarized intercalated disk	[71]
Nucleation	~25% bi/multinucleated in adult human myocardium	Predominantly mononucleated; ~17% bi/multinucleated has been documented	[72,73,77,78]
Mitochondria	~30–40% of cell volume; dense cristae; uniform distribution; coupled to sarcoplasmic reticulum (SR)	Low volume fraction; irregular distribution; poorly organized cristae	[70,73]
**B. Electrophysiological**			
**Parameter**	**Adult Human CM**	**hiPSC-CM (Standard 2D)**	**Reference(s)**
Spontaneous activity	None; stimulus-dependent only; HCN4 largely restricted to pacemaker cells	Spontaneous beating; mixed AP morphologies (atrial/nodal/ventricular-like); HCN4 broadly expressed	[70,73,79,80]
Resting membrane potential (RMP)	−80 to −90 mV; IK1-stabilized	−20 to −60 mV; IK1 absent or markedly reduced	[75,79]
Upstroke velocity	~150–350 V/s	~10–50 V/s	[28,75]
Conduction velocity	~60 cm/s	~10–20 cm/s	[28,75]
Repolarizing currents	IKr and IKs; IK1 stabilizes RMP	IKr and IKs present; IK1 absent or markedly reduced	[79]
**C. Mechanical/Contractile**			
**Parameter**	**Adult Human CM**	**hiPSC-CM (Standard 2D)**	**Reference(s)**
Force generation	~μN range (single cells) ~40–80 mN/mm^2^ (muscle strips)	~200 nN (single cells) ~0.08–4 mN/mm^2^ (3D constructs)	[28,75]
Force–frequency relationship	Positive (Bowditch effect)	Negative or absent	[81,82,83]
β-adrenergic responsiveness	Robust chronotropic, lusitropic, and inotropic response	Present but not as robust—immature; limited in early (20–40 d) cultures; improves with prolonged culture	[31,84]
**D. Calcium Handling**			
**Parameter**	**Adult Human CM**	**hiPSC-CM (Standard 2D)**	**Reference(s)**
Ca^2+^ release mechanism	CICR via T-tubule L-type channels/ryanodine receptor 2 (RYR2) dyads; synchronized; ~70% Ca^2+^ removal by SERCA2a	L-type channel-dependent (no T-tubules); less coordinated Ca^2+^ release due to increased distance between L-type Ca^2+^ channels and RyR2; increased fraction of cytosolic Ca^2+^ removed via Na^+^/Ca^2+^ exchanger (NCX)	[85,86,87]
SR/SERCA2a	Well-developed SR; high expression level of sarco/endoplasmic reticulum Ca^2+^-ATPase 2a (SERCA2a) and calsequestrin (CASQ2)	Less structurally mature SR network; lower expression of SERCA2a, CASQ2, RYR2, phospholamban (PLN) in early/standard 2D culture, with expression increasing with maturation over time and in engineered tissues	[79,85]
**E. Metabolic**			
**Parameter**	**Adult Human CM**	**hiPSC-CM (Standard 2D)**	**Reference(s)**
Primary energy substrate	FAO dominant; oxidative phosphorylation is the major ATP source; glycolysis minor contribution	Glycolysis dominant; immature mitochondrial oxidative metabolism; FAO capacity low and increases with maturation; fetal-like metabolic profile	[38,73,88]
Glucose transporters	GLUT4 (SLC2A4) dominant (insulin-responsive); GLUT1 relatively lower contribution	GLUT1 (SLC2A1) highly expressed; GLUT4 low/immature; limited or immature insulin-responsiveness	[88,89]
**F. Molecular/Isoform**			
**Parameter**	**Adult Human CM**	**hiPSC-CM (Standard 2D)**	**Reference(s)**
Troponin I isoform	TNNI3 (cTnI) dominant; TNNI3:TNNI1 ratio = maturation index, irreversible change ‘benchmark’	TNNI1 (ssTnI) dominant; fetal-like ratio	[59]
Myosin heavy chain	β-MHC (MYH7) is dominant; α-MHC (MYH6) present at ~5–10%; dynamic and reversible in disease states	Immature myosin isoform profile with little or no adult α-MHC (MYH6) expression; α-MHC reported low or absent	[7,71]
Titin isoform	N2B dominant (stiff, short, adult)	N2BA dominant (compliant, longer, fetal)	[71,73,75,90]
PDE isoform	PDE3 dominant	PDE4 and PDE3 with predominance to PDE4	[8]
Hyperpolarization-activated cyclic nucleotide gated 4 (HCN4) channel	HCN4 largely restricted to pacemaker cells	HCN4 broadly expressed; drives spontaneous beating	[70,73,79,80]
SERCA2a, caveolin-3 (CAV3), potassium voltage gated channel subfamily h member 2 (KCNH2)	High expression	Low expression compared to adult CM	[28,79]

## 4. Induced Maturation in 2D Monolayers

**Figure 3:**
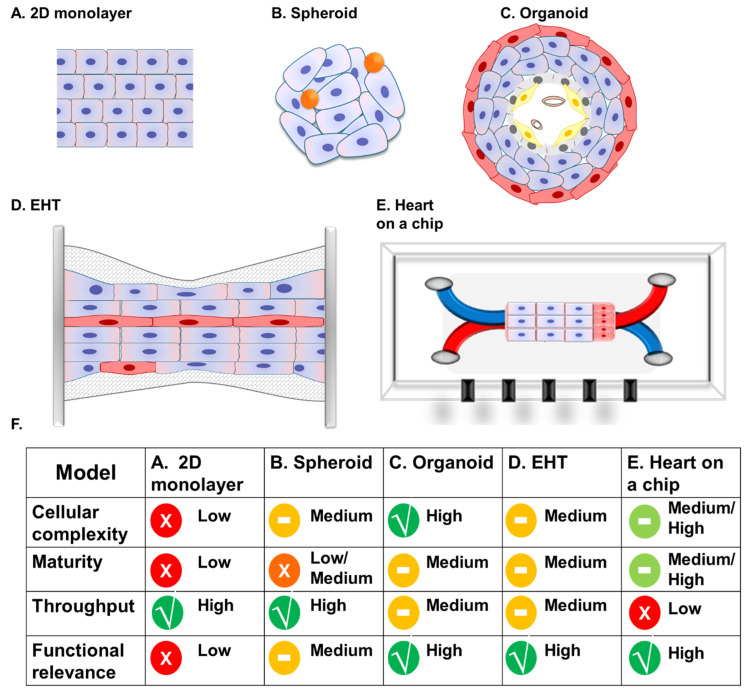
Overview of in vitro human cardiac tissue models. (**A**) 2D monolayer culture of cardiac myocytes, showing flattened cardiac myocytes (purple) grown on rigid substrates. (**B**) Cardiac spheroid, a 3D aggregate of cell populations (mainly cardiac myocytes (purple) and fibroblasts (orange–red)). (**C**) Cardiac organoid, illustrating a more complex, self-organized structure with multicellular composition and spatial patterning, including cardiac myocytes (purple), fibroblast (orange–red), stromal cells (gray), endothelial cells (yellow), and vascular-like compartments (beige) surrounding a central lumen like region. (**D**). Engineered heart tissue (EHT), consisting of aligned cardiac myocytes (purple), and fibroblasts (orange–red) embedded within a biomaterial scaffold and anchored between flexible posts, enabling force generation and mechanical loading. (**E**). Heart on a chip, showing a microfluidic platform containing engineered cardiac tissue connected to perfusion channels (red/blue) with integrated sensors, enabling controlled flow, electrical and mechanical stimulation, and real-time functional readouts. (**F**). Table highlighting main points of comparison between models.

2D monolayer systems remain a foundational platform for studying hiPSC-CM maturation due to their experimental accessibility, scalability, and compatibility with high-throughput assays (Figure 3). They offer an excellent platform for dissecting individual maturation drivers. Bedada et al. established that immature sarcomeric architecture, characterized by disorganized sarcomere, and fetal isoform predominance, represents a central bottleneck in hiPSC-CM maturation, as intrinsic developmental programs remain incomplete without external instructive cues [26,59].

Engineered substrate cues play a central role in driving early cytoskeletal and functional maturation. Geometric confinement using micropatterned substrates enforces physiological elongation (aspect ratios ~7:1), promoting anisotropic organization and improving contractile function [91,92,93,94]. Substrate stiffness is a critical regulator of cardiac myocyte maturation. Matrices tuned to physiological myocardial stiffness (~10 kPa) promote optimal sarcomere organization, contractile force generation, and electrophysiological properties, whereas excessively rigid substrates impair myofibrillar structure and reduce functional output [91,95,96]. This effect is mediated through integrin-FAK signaling, RhoA/ROCK activation, and downstream regulation of YAP/TAZ mechanotransduction, linking extracellular mechanics to transcriptional maturation programs [97].

Substrate biochemical composition further modulates hiPSC-CMs maturation. The cardiac-derived extracellular matrix (ECM) provides tissue-specific biochemical cues, including collagen and glycosaminoglycans, to enhance sarcomere organization and calcium handling and to support metabolic and functional maturation. However, these systems induce only partial maturation and do not fully recapitulate adult myocardium transcriptional or metabolic states [98,99,100]. In addition to passive substrate properties, dynamic biophysical stimulation significantly enhances maturation. Electrical pacing promotes synchronization, improves action potential characteristics, and enhances calcium cycling, while mechanical loading reinforces sarcomeric alignment and force transmission [101,102,103,104]. Directed genetic and transcriptional programming drives maturation by promoting adult sarcomeric isoforms (as noted above with MYH and TNNI isoforms) and suppressing fetal gene programs [7,26,50,59,68,105,106].

Metabolic and hormonal interventions further drive maturation toward an adult-like cardiac phenotype. Supplementation with thyroid hormone and glucocorticoids, alongside metabolic switching to fatty acid oxidation, enhances mitochondrial function, electrophysiological properties, and contractile performance [73,107]. Prolonged culture also contributes to progressive sarcomeric organization and increased functional output, although maturation remains incomplete relative to adult cardiac myocytes [31].

Despite these advances, several features consistently impede maturation in 2D systems: (i) absence of transverse tubules and dyadic coupling, limiting fully synchronized Ca^2+^ release; (ii) incomplete intercalated disk formation and mechanical junctional integrity; (iii) persistent fetal electrophysiological phenotype; and (iv) incomplete metabolic specialization with residual glycolytic flux. These limitations arise from the lack of physiological load, spatial dimensionality, vascularization, and multicellular niche signaling.

While 2D platforms remain indispensable for mechanistic and screening studies, they are best understood as tools that amplify individual maturation cues rather than achieving full adult maturity, motivating the ongoing experimental platform transition to multicellular and higher-dimensional models.

## 5. 3D Cultures: Architecture and Cell Fate

Cardiac function is inextricably linked to the exquisite alignment of the cardiac myocytes (CMs) within the heart. This highly ordered architecture enables rapid electrical conduction and coordinated, forceful contraction [108]. Reproducing this architectural organization is therefore central to engineering physiologically relevant hiPSC-CM models. Single-cell traction force microscopy studies demonstrate that geometry, stiffness, and prolonged culture markedly enhance force generation and electrophysiological maturity [96], demonstrating that mechanical context functions as a biological signal, providing a mechanistic foundation for why 3D platforms produce progressively more mature phenotypes.

Patient-specific iPSCs have been used to model inherited disorders such as long QT syndrome, hypertrophic cardiomyopathy, and dilated cardiomyopathy [109,110,111]. They have also been used to model non-genetic conditions including ischemia, hypertrophy, cancer cachexia-associated cardiomyopathy, and cardiotoxicity [3,17,112,113,114,115]. However, here 2D systems remain limited. Monolayer cultures lack the structural complexity of native cardiac tissue, restricting paracrine signaling and intercellular crosstalk. Cardiac myocyte maturity in 2D remains relatively low (Table 1). Functional measurements are often confounded by cellular heterogeneity, leading to variable contractile outputs compared with the more integrated and reproducible readouts of 3D microtissues. Additionally, 2D models primarily capture direct drug effects on cardiac myocytes and fail to account for systemic interactions such as hepatic metabolism, underscoring the need for multi-organ, perfused platforms [3,116,117,118].

To address this need, cardiac tissue engineering has increasingly shifted toward 3D cellular constructs, such as spheroids and organoids, which provide microenvironments that address geometry, mechanical cues, enhance cell–cell interactions and promote CM maturation [119]. 3D cultures function as engineered niches in which three-dimensional geometry acts as a biological signal influencing cell–cell coupling, mechanical integration, and maturation state [120,121], enhancing structural and functional fidelity, and enabling enhanced physiologically relevant disease modeling. These systems increasingly incorporate fibroblasts, endothelial cells, and other supporting populations to better reflect myocardial complexity [3,37,122,123,124,125,126,127,128,129]. A wide range of platforms have emerged, including spheroids, organoids, engineered heart tissues (EHTs), and heart-on-chip systems [3,37,122,123,124,125,126,127,128,129].

Over the last decade, 3D cardiac spheroids, organoids, and engineered heart tissues (Figure 3) have demonstrated that spatial context reprograms phenotypes [78]. Transcriptomic comparisons show that 3D environments upregulate extracellular matrix (ECM) organization, angiogenic pathways, and cell–cell communication programs compared with 2D cultures [37,130,131,132,133]. Multicellular human cardiac organoids (hCOs) incorporating fibroblasts and endothelial cells show greater similarity to adult human myocardium than 2D or monocellular 3D CM-only aggregates, although they still cluster closer to fetal myocardium when benchmarked against bulk RNA-seq datasets of adult ventricles [37,130,131,132,133].

### 5.1. Cardiac Spheroids: Minimal 3D Microtissues for Cell–Cell Coupling

Cardiac spheroids represent the simplest transition from two-dimensional culture to three-dimensional cardiac tissue organization (Figure 3). Generated by the self-aggregation of cardiac myocytes alone or in combination with fibroblasts and endothelial cells, spheroids enable direct cell–cell coupling, paracrine signaling, and diffusion gradients that are absent in monolayer systems [126,134,135]. This geometry markedly influences cell fate, with spheroid-cultured cardiac myocytes exhibiting improved sarcomere organization, enhanced calcium handling, and more physiologically relevant electrophysiology compared with 2D cultures, reflecting the role of tissue geometry as a biological signal [126,134,135]. Importantly, spheroids allow systematic control of size and cellular composition, enabling high-throughput investigation of multicellular interactions, metabolic gradients, and drug responses [126,134,135]. Spheroids are therefore best understood as controlled, scalable platforms for studying early tissue assembly, multicellular signaling, and initial maturation cues.

### 5.2. Cardiac Organoids

Cardiac organoids can be viewed as an extension of spheroid systems, representing the attempt to compress the multicellular, mechanically loaded and metabolically demanding complexity of the native heart into a controllable human platform. The key distinction between spheroids and organoids lies not in size or cell number but developmental and biological complexity. Cardiac organoids employ multilineage developmental signaling, self-organization, or bottom-up multicellular assembly, and morphogen-guided regulation to generate structures with emergent tissue architecture, regional identity, or vascular components. Spheroids integrate maturation cues and multicellular interactions in a more controlled, reductionist format, whereas organoids attempt to recapitulate aspects of cardiogenesis, tissue-level organization, and higher-order disease biology [37,125,126,131,132,133,134,135,136,137,138,139,140,141,142,143].

Mechanistically, this shift toward greater biological complexity is enabled by developmental programming. Cardiac organoids leverage the multilineage differentiation capacity of hiPSCs to generate structured cardiac tissue [136]. Efficient organoid generation mirrors embryonic cardiogenesis [144]. Pluripotent cells traverse cardiogenic mesoderm and cardiac progenitor states under tightly choreographed signaling. Canonical Wnt/β-catenin activation (e.g., CHIR99021) followed by inhibition (IWP2, IWP4, Wnt-C59) remains the backbone of cardiac myocyte differentiation. Temporal control of bone morphogenetic protein (BMP), activin/nodal, fibroblast growth factor (FGF), and retinoic acid (RA) signaling tunes lineage allocation and chamber identity. RA gradients, for example, bias atrial versus ventricular specification, which is particularly relevant for regionally patterned organoid design. Self-organization approaches, including “cardioids,” introduce morphogenetic processes that drive emergent structure formation [23,136].

In the landmark work by Hofbauer et al. [125] temporal modulation of Wnt, BMP, activin, FGF, and RA signaling yielded cavity-containing cardiac structures without exogenous ECM [125,137]. This demonstrated that intrinsic patterning programs alone are sufficient to generate chamber-like cavities, albeit without full epicardial complexity. Subsequent simplified approaches reduced growth factor complexity while preserving cavity formation, underscoring how much morphogenetic information is encoded in developmental timing [129]. The implication is fundamental: tissue structure is itself a signaling output.

Human cardiac organoids can be formed via self-organization, developmental patterning, or bottom-up assembly of defined cardiac populations. Patterning approaches generate “cardioid” models that recapitulate early cardiogenesis, while multicellular assembly and biofabrication strategies improve tissue organization, vascular features, and adult-like transcriptomic profiles. Across platforms, maturation gains must be balanced against trade-offs between reproducibility and developmental fidelity [131,133,136,138,139,140,141,142,143].

Cardiac organoids enable multicellular crosstalk among cardiac myocytes, fibroblasts, and endothelial cells, driving structural, electrophysiological, and transcriptional maturation beyond 2D systems [37]. Cardiac organoids’ 3D architecture supports tissue-level phenotypes, including ECM remodeling and inflammatory responses seen in cardiac injury and drug responses in a context that includes stromal and vascular components [131], while remaining well-suited for developmental and congenital disease modeling, where tissue organization and lineage interactions drive morphogenesis [125,137]. With increasing scalability, cardiac organoids provide a powerful platform for modeling human cardiac development, injury, and therapeutic response [145,146,147,148].

However, even the best human cardiac organoids resemble fetal myocardium more than adult ventricle at the transcriptomic level. Metabolic programming (glycolytic bias), sarcomeric organization, t-tubule formation, and force–frequency relationships remain incomplete [78,131,133,136,137,144]. In addition, cellular diversity is incomplete. Immune cells are typically absent, despite their central role in cardiac remodeling [149]. Age is another factor. HiPSC-derived fibroblasts resemble embryonic fibroblasts, and aging signatures profoundly influence fibroblast heterogeneity and myocardial mechanics [150,151]. Adult primary fibroblasts may enhance cellular heterogeneity, but at the cost of genetic mismatch and scalability. Diffusion limits impose metabolic gradients that present physiological challenges. Moreover, long-term maintenance of non-CM populations is tenuous. Some organoid systems progressively lose non-myocytes, revealing that medium composition is a selective pressure on organoid stability [132,136].

Cardiac organoids should be viewed as tunable systems balancing complexity and control. By adjusting developmental signaling, cellular composition, ECM context, and biomechanical stimulation, researchers can shift organoids along a spectrum from simplified mechanistic models to more physiologically representative tissues [37,125,131,132,136,137]. The next phase of platform development therefore lies in combining multicellular complexity with metabolic competence, immune integration, and spatially resolved omics.

### 5.3. Engineered Heart Tissues (EHTs)

Engineered heart tissues (EHTs) sit in the sweet spot where cardiac bioengineering and biology meet to transfer the hiPSC-CM monolayer to 3D. EHT are developed to restore the core features that characterize functional myocardium: force generation, synchronized cell–cell coupling, electromechanical integration, and the emergence of diffusion gradients that simply cannot exist in 2D culture.

EHTs are generated by combining pre-differentiated cardiac myocytes with supportive non-cardiac myocytes within a hydrogel matrix, typically cast into defined molds and anchored to elastic supports that impose mechanical load (Figure 3) [152,153,154,155,156,157,158]. This mechanical loading is one of the most critical determinants of tissue formation, cardiac myocyte alignment, and force production. By imparting aspects of myocardial load and architecture, EHTs promote longitudinal organization of cells along force-bearing axes, yielding a 3D construct capable of coordinated contraction [152]. Matrix and scaffold optimization (e.g., fibrin hydrogels, elastic posts) enable auxotonic contraction, allowing tissues to perform work against resistance rather than contracting under unloaded conditions [153,154,155,156,157,159,160]. The integration of electrical or optical pacing further enhances maturation and functional output of EHT [152,161,162,163].

Functionally, EHTs enable aligned syncytial contraction with direct measurement of force and kinetics [153,157,160], referred to as “in vitro echocardiography,’’ and provides steady-state functional monitoring over weeks [153]. EHT also supports multicellular conduction pathways and pacing responsiveness [164]. Despite these advantages, key immaturities persist, including incomplete t-tubules development and diffuse connexin-43 localization [154,160,164]. These observations highlight a recurring theme in the field: EHT raises the maturation ceiling substantially but does not yet reach a fully adult state.

Multicellular composition in EHT further enhances performance, notably by incorporation of fibroblasts and endothelial cells improves compaction, ECM remodeling, and vascular-like structures [37,102,159,165]. Tiburcy et al. demonstrated that fibroblasts are particularly important for hydrogel condensation and optimal force generation, with ~70% cardiac myocytes and ~30% fibroblasts emerging as a useful ratio [165]. More recent immune-integrated constructs, such as macrophage-containing EHT, further expand disease modeling fidelity, including myocarditis-like inflammatory dysfunction [166]. Ronaldson-Bouchard et al. showed that progressive electrical pacing up to physiological frequencies can drive striking ultrastructural improvements, including near-adult sarcomere lengths and enhanced calcium handling [102]. Still, even these advanced systems reveal persistent limitations in metabolic and electrophysiological development, underscoring that maturation is multi-dimensional, not a single endpoint.

From a translational perspective, EHT is well positioned as a scaffold-based system for drug screening, cardiotoxicity testing, and patient-specific disease modeling. They can reproduce expected responses to ion channel blockers, β-adrenergic stimulation, and afterload stress, and they are increasingly used to model inherited cardiomyopathies and channelopathies [153]. EHT stability also enables long-term interventions, making them particularly valuable for compounds with delayed structural or electrophysiological effects.

However, the EHT platform comes with some challenges. EHT requires relatively high cell numbers, extended culture times before functional readout, and careful control of differentiation efficiency and cellular composition. Reproducibility across hiPSC lines remains a limitation, and scalability is still largely limited to low or medium throughput formats [153,167,168]. Moreover, while EHTs outperform 2D cultures in terms of functional maturity, their contractile reserve, β-adrenergic responsiveness, calcium handling dynamics, and metabolic switching reflect non-adult-like phenotypes, reinforcing the concept of a “maturation ceiling” that the field continues to contend with.

Overall, EHT represents a physiologically relevant intermediate between 2D cultures and native myocardium, providing a powerful experimental arena for studying cardiac function, while still carrying the reproducibility, scalability, and maturation gaps that define the next frontier of cardiac tissue engineering.

### 5.4. Heart-on-a-Chip Platform: Engineered Microphysiology and Electromechanics

Heart-on-a-chip systems extend cardiac tissue engineering into microphysiological systems that integrate tissue architecture with controlled mechanical and electrical environments modeling [169,170,171,172,173,174,175,176]. Heart-on-a-chip devices typically combine microfabricated scaffolds, perfusion channels, and engineered cardiac tissues to reproduce key aspects of myocardial physiology, including force generation, electrical conduction, and biomechanical loading. Unlike self-organizing organoids, heart-on-a-chip systems incorporate precise structural control and reproducibility, enabling quantitative measurements of contractile force, electrophysiology, and drug responses under defined mechanical conditions. This engineering-driven architecture supports long-term maturation and allows integration with vascular flow or multi-organ microfluidic networks, making these systems particularly valuable for pharmacology and disease modeling [169,170,171,172,173,174,175,176]. However, their reliance on predefined scaffolds can limit the spontaneous tissue patterning observed in organoids, highlighting a conceptual tradeoff between biological self-organization and engineering control. Together with spheroids, organoids, and engineered heart tissues, heart-on-chip technologies illustrate how three-dimensional geometry, multicellular composition, and electromechanical context collectively shape cardiac myocyte identity and cardiac tissue function.

Balancing biological complexity with scalability is particularly relevant for 3D systems. Their deployment at the industrial scale is constrained by high cell number requirements, extended culture timelines of several weeks before functional readout, batch-to-batch variability driven by differentiation efficiency, and the technical demands of tissue fabrication [152,167,168]. For high-throughput drug screening specifically, these limitations are not trivial. Cardiotoxicity assessments require reproducible, quantifiable functional readouts across hundreds to thousands of compounds, a throughput that current EHT and organoid formats do not yet support in standard laboratory settings. Emerging strategies are beginning to address this gap: miniaturized EHT formats in 96-well configurations enable simultaneous contractile/Ca^2+^ measurements. Additionally, suction-assisted microwell platforms can generate tens of thousands of uniform cardiac spheroids/organoids per run, and automated imaging and impedance-based readout systems increasingly reduce operator-dependent variability [77,145,146,147,148,152,167,168,177,178]. Increasing numbers of organoid centers and commercial platforms world-wide are driving the next generation of scalable, reproducible, and clinically relevant human tissue modeling systems. These advances notwithstanding, fully reconciling biological fidelity with industrial scalability remains an unresolved engineering and biological challenge.

## 6. Cardiac Slices as Living Scaffolds for Maturation

Native human cardiac slices offer structural and biochemical fidelity that synthetic 2D and 3D systems cannot fully replicate. Ultra-thin sections of native myocardium (<400 μm) preserve anisotropic ECM, vascular remnants, and localized signaling cues [179], functioning as “living scaffolds” that bridge simplified in vitro models and in vivo transplantation. With optimized media [e.g., insulin–transferrin–selenium (ITS), fibroblast growth factor (FGF)/vascular endothelial growth factor (VEGF)] and electrical stimulation, slices maintain contractile force and calcium homeostasis for several days [180]. Interestingly, these native models rely on glycolysis, a hallmark of immature hiPSC-CMs, suggesting that the in vitro environment itself imposes constraints on oxidative phosphorylation [180,181]. This metabolic flexibility (FAO to glycolysis), however, provides a unique testing ground for hiPSC-CMs to integrate and mature within a physiologically relevant environment.

Decellularized human cardiac slices retaining key ECM components (collagen I/III, laminin) serve as high-fidelity scaffolds for cell seeding in engineered heart slices (EHS) [59,182]. Our group has previously demonstrated successful decellularization and subsequent recellularization of cardiac slices, highlighting the potential of this platform for reconstructing multicellular cardiac tissue with preserved ECM organization [59]. Additionally, recellularization platforms demonstrate remarkable phenotypic instruction. First, seeded hiPSC-CMs exhibit elongated morphology and aligned sarcomeres that persist in long-term culture (up to 9.5 months) [59,182]. Additionally, EHS models show uniform conduction and anisotropic action potential propagation, mimicking native tissue behavior [182]. However, despite these structural and electrophysiological improvements, a maturation gap remains. EHS exhibit lower fractional shortening (2% vs. 30% in adults) and a negative force–frequency relationship, indicating that ECM alone is insufficient for full functional maturation [182].

## 7. Multicellular Reconstitution: Fibroblasts, Endothelial Cells, Immune and Neural Inputs

The native human myocardium is composed of multiple interacting cell types that collectively regulate structural organization, electrophysiology, and metabolic function [37,183]. Cardiac myocyte maturation is therefore a non-cell-autonomous process, emerging from bidirectional signaling between cardiac myocytes and non-myocyte cell populations. Incorporation of cardiac fibroblasts and endothelial cells enhances structural and functional maturation. Fibroblasts contribute to extracellular matrix deposition and remodeling, modulating tissue stiffness and promoting anisotropic organization of cardiac myocytes [183,184]. Beyond structural roles, fibroblasts regulate cardiac myocyte maturation through transforming growth factor beta (TGF-β), periostin, and ECM-integrin signaling axes, which influence cytoskeletal tension, sarcomeric assembly, and electrophysiological coupling [185]. In experimental models, fibroblasts of cardiac origin can establish gap junction-mediated electrotonic coupling with cardiac myocytes over extended distances [186], though the functional significance of this coupling in human myocardium remains to be established. The functional impact depends on fibroblast density, age and coupling strength. In silico studies suggest that fibroblast density exerts a biphasic effect on conduction velocity, where modulating fibroblast content influences conduction block and arrhythmogenesis, highlighting the need for controlled cell stoichiometry in engineered tissues [187].

Endothelial cells provide paracrine signals, including nitric oxide, VEGF, and neuregulin-1 that improve contractility, calcium handling, and electrophysiological properties [188,189]. Collectively, multicellular co-culture improves sarcomeric organization, connexin-43 expression, mitochondrial maturation, electrical coupling, and force generation, yielding a more physiologically relevant myocardial phenotype [37].

Immune cells, particularly cardiac macrophages, modulate cardiac myocyte function and tissue homeostasis. They form connexin-43 gap junctions with cardiac myocytes particularly in the distal AV node, facilitating electrical conduction [190,191,192]. This macrophage–cardiac myocyte electrical coupling represents a paradigm shift, demonstrating that non-excitable cells can directly regulate impulse propagation. Beyond this, macrophages secrete paracrine factors such as interleukins and nitric oxide that influence cardiac myocyte survival, electrophysiology, and remodeling processes [191,193]. Incorporation into engineered tissues alters inflammatory signaling and enhanced structural organization, suggesting that controlled immune signaling may accelerate maturation but requires tight regulation to avoid pathological remodeling phenotypes [194,195].

Emerging studies also demonstrate the importance of neuronal inputs in regulating cardiac myocyte behavior. Functional co-culture of either hiPSC-derived neurons or murine sympathetic neurons with hiPSC-derived cardiac myocytes reveals electrophysiologically functional neuron–CM interactions [196,197]. Sympathetic innervation further promotes maturation, introducing physiological neurohumoral regulation absent in monoculture systems [196].

Together, cardiac myocyte maturation emerges as a systems-level process driven by ECM and mechanical cues (fibroblasts), angiocrine and metabolic signaling (endothelium), immune modulation (macrophages), and neurohumoral input (neurons), collectively coordinating sarcomeric organization, electrophysiology, metabolism, and stress responses.

## 8. Vascularization: From Diffusion to Perfusion

In native myocardium, most cells reside within a few hundred micrometers of a capillary; beyond this distance, diffusion is insufficient to sustain viability [198,199,200]. Engineered myocardium exceeding ~1 mm in thickness therefore requires vascular networks to support mass transport [201,202].

To address this limitation, several vascularization strategies have been developed. Co-culture systems incorporating endothelial cells and supporting mural cells can promote spontaneous microvascular network formation within engineered cardiac tissues [199,203]. Scaffold architecture and extracellular matrix properties further regulate vascular morphogenesis, while microfabrication and microfluidic approaches enable the generation of predefined perfusable channels that mimic vascular flow environments [169,200,204]. Growth factor delivery and perfusion bioreactors are also commonly employed to guide angiogenesis and vascular maturation [198,205].

Importantly, the transition from diffusion limited constructs to perfused systems significantly alters the microenvironment of engineered myocardium. Perfusion improves oxygen and nutrient transport, enhances spatial cell distribution, and promotes cardiac tissue organization and phenotype compared to static culture conditions [198,206]. Recent work using hiPSCs further demonstrates that pre-vascularized engineered cardiac tissues containing endothelial networks exhibit enhanced survival and vascularization along with improved functional performance and integrated with host vasculature following implantation, forming perfused hybrid capillary networks [207].

In addition, vascularized microphysiological systems allow drug exposure from a vascular compartment, enabling more realistic pharmacokinetic and pharmacodynamic interactions while facilitating the study of endothelial–cardiac myocyte crosstalk and vascular barrier function [208]. Together, these advances highlight vascularization as a critical determinant of long-term maturation, tissue stability, and translational fidelity in engineered cardiac systems.

## 9. Integrative Maturation: Convergence of Multiscale Cues

The persistent immaturity of hiPSC-CMs reflects a fundamental limitation in how maturation has been historically conceptualized, namely, as a series of modular, independently optimizable processes. In contrast, cardiac maturation in vivo is neither linear nor compartmentalized; it is a tightly orchestrated, multiscale phenomenon emerging from the convergence of transcriptional programs, electrophysiological and biomechanical forces, metabolic transitions, environmental and tissue-level architecture (Figure 1). Reframing maturation as a systems-level outcome rather than a discrete endpoint provides a more faithful biological and engineering paradigm.

Past maturation efforts focused on isolated perturbations, electrical pacing, hormonal stimulation, substrate stiffness, or metabolic rewiring, each producing partial gains [73,88,102,181]. These interventions do not, however, operate additively. Instead, they exhibit nonlinear interdependencies, where the presence or absence of one cue fundamentally alters the cellular response of another. This is consistent with developmental biology principles, where emergent phenotypes arise from coupled feedback loops across scales, rather than from single dominant regulators.

Molecular changes appear inherently unstable or incomplete in the absence of functional demand. Electromechanical conditioning, through chronic pacing and afterload, provides a necessary stabilizing context, reinforcing sarcomeric alignment and excitation-contraction coupling [102,209]. This suggests that transcriptional maturation must be “locked in” by biomechanical utilization, echoing the use-dependent maturation observed in postnatal myocardium. Metabolic maturation constitutes another essential dimension. While supplementation with fatty acids or PPAR agonists can induce aspects of metabolic remodeling, these interventions are constrained by environmental limitations, most notably oxygen diffusion and substrate delivery in static cultures [63,88,181,210]. In this context, vascularization and perfusion emerge as enabling infrastructures. Perfused micro-physiological systems restore the coupling between metabolic demand and supply, allowing sustained oxidative metabolism and preventing hypoxic drift, thereby stabilizing mitochondrial and electrophysiological maturation [206,211,212,213].

3D organization further introduces a higher-order regulatory layer, restoring anisotropic alignment, physiological force transmission, and cell–cell coupling, facilitating reciprocal feedback between structure and function [133,165]. Within these constructs, the inclusion of non-myocyte populations; cardiac fibroblasts, endothelial cells, and perhaps immune or neural components, recapitulates key aspects of the myocardial niche.

A critical component of integrative hiPSC-CM maturation is the metabolic transition from glycolytic flux to fatty acid oxidation (FAO), a process that is often incomplete in standard culture conditions. While conventional media (e.g., RPMI-B27^+^) provide high glucose levels that sustain a fetal-like glycolytic state, specialized maturation media, including both commercial formulas and those described in the literature, aim to better recapitulate the physiological postnatal environment by substituting glucose with galactose, supplementing with albumin-bound fatty acids (e.g., palmitate, oleate, and linoleate), thyroid hormone (T3), dexamethasone, and PPARα agonists [181,210,214,215]. However, a significant discrepancy remains between these in vitro formulations and the complex, dynamic physiological milieu. Most maturation media are designed for endpoint maturation rather than developmental progression, often lacking the precise hormonal orchestration, the physiological calcium level, and fatty acid proportions found in vivo [26,59,64,210,214]. Furthermore, while these media successfully enhance mitochondrial biogenesis and FAO capacity, they may not fully capture the metabolic flexibility required for adult-like responses to stress or pathology [214,215,216]. Addressing this gap requires a move toward media that integrates metabolic substrates with systemic endocrine dynamics to ensure that hiPSC-CMs are not only metabolically switched but also physiologically resilient.

## 10. Gaps and Controversies

The potential application of mature hiPSC-CM systems for the interpretation of variants of uncertain significance (VUS) in cardiovascular disease deserves consideration [217]. With the widespread adoption of next generation whole genome sequencing, an expanding catalog of VUSs has been identified across sarcomeric, ion channel, and desmosomal genes associated with heritable cardiomyopathies and channelopathies [218,219,220]. One of the major limitations of immature hiPSC-CMs is that fetal-like electrophysiological, metabolic, and structural phenotypes can obscure subtle genotype–phenotype relationships, thereby reducing the predictive value of disease modeling and functional genomics studies. Thus, advanced maturation is a prerequisite for clinically meaningful precision medicine applications, including VUS classification, patient stratification, and drug response prediction [73,221].

Whether full adult myocardial maturation is achievable in vitro remains unresolved. While advanced systems recapitulate many structural and functional features, complete convergence with adult myocardium, particularly in terms of long-term stability, electrophysiological precision, and metabolic flexibility, has not been definitively demonstrated.

A central limitation is the absence of systemic regulation. In vivo, cardiac myocyte maturation is orchestrated by endocrine signals, neural input, and hemodynamic forces that fluctuate over time. Replicating these dynamic and hierarchical cues in vitro remains a formidable challenge. Furthermore, current models often exhibit heterogeneity in maturation states, complicating interpretation and reproducibility.

The inherent genetic variability between independent hiPSC lines represents an important source of functional heterogeneity that can significantly impact the efficacy and reproducibility of maturation protocols [222]. Line-to-line differences in genetic background have been shown to influence transcriptional states, differentiation efficiency, and electrophysiological or metabolic trajectories meaning that a maturation protocol optimized for one line may produce substantially different outcomes in another [223]. This necessitates careful consideration of genetic background effects and, where possible, the inclusion of multiple hiPSC lines to ensure robustness and generalizability of findings.

Transplantation studies provide important insights, as hiPSC-CMs introduced into in vivo environments undergo further (often partial but progressive) maturation [224,225,226]. However, this raises a critical question: does in vivo maturation reflect intrinsic cellular potential or extrinsic environmental instruction that cannot be fully replicated ex vivo? Another area of debate concerns the definition of “maturity.” Different studies prioritize structural, functional, or molecular endpoints, leading to inconsistent benchmarks and claims of maturity. This underscores the need for standardized, multi-dimensional criteria for cardiac maturation in vitro, including composite scores that reflect the integrated nature of cardiac myocyte biology (Table 1, Figure 2).

## 11. Defining the Maturation Ceiling

A robust framework should incorporate multi-dimensional indices encompassing structural, functional, molecular, and metabolic parameters. Rather than relying on single metrics, maturation should be quantified using composite scoring systems that reflect the integrated nature of cardiac myocyte biology. For example, combining sarcomere organization, force generation, calcium handling kinetics, and isoform expression provides a more comprehensive assessment than any individual measure. While the field has not yet established formally validated consensus thresholds, the present literature supports the following provisional reference values drawn from primary human myocardial data (Table 1 and Figure 2) as working benchmarks against which in vitro systems should be assessed. These benchmarks include cell aspect ratio ~7:1; sarcomere length ~2.0 µm; resting membrane potential of −80 to −90 mV, with IK1-stabilized; force generation ~40–80 mN/mm^2^ for a muscle strips; TNNI3:TNNI1 protein isoform ratio approaching adult levels (i.e.,TNNI3 (cTnI) nearly 100%); dominant β-MHC with α-MHC at ~5–10%, adult CM’s T-tubule density; organization and calcium handling; mitochondrial volume (>30% of cell volume); fatty acid oxidation being the dominant energy source; and a positive force–frequency relationship [7,26,28,31,38,57,58,70,71,73,74,75,76,79,81,82,83,85,86,87,88]. No current in vitro system simultaneously achieves all these benchmarks, and this coordinated deficit across parameters, rather than any single deficit, operationally defines the maturation ceiling. The composite maturation index should weigh these parameters according to the application. For example, electrophysiological fidelity is critical for arrhythmia modeling, while metabolic competence is prioritized for cachexia, ischemia or heart failure studies, and force generation is central for regenerative therapy evaluation.

The use of reference standards derived from human myocardium across developmental stages is valuable for direct comparison between in vitro models and in vivo counterparts. Advances in single-cell and spatial transcriptomics offer opportunities to define molecular “age” signatures, which can serve as quantitative benchmarks for maturation. Single-cell RNA sequencing (scRNA-seq) provides high-resolution insights into cellular heterogeneity and maturation trajectories in hiPSC-CMs, although it is limited by the loss of native tissue architecture and intercellular interactions following tissue dissociation [227]. In contrast, spatial transcriptomics preserves the structural integrity of EHTs or organoids, allowing for the mapping of maturation gradients and niche-specific interactions, though often at the cost of lower cellular resolution and higher technical complexity [228,229]. Together, these technologies offer a quantitative framework for standardizing maturation assessment and establishing reference benchmarks for hiPSC-CM maturation.

Standardization should also extend to experimental design and reporting, including cell sourcing, culture conditions, stimulation protocols, and analytical methods. The establishment of community-driven guidelines and shared datasets will facilitate reproducibility and accelerate progress.

## 12. Conclusions: Standardizing Benchmarks and Roadmaps

High-fidelity integrative systems combining genetic isoform control with environmental cues remain largely unexplored, highlighting a critical gap. The field would benefit from a model in which maturation arises from interdependent coupling between molecular, metabolic, and biomechanical inputs rather than isolated interventions. This convergence necessitates a conceptual shift toward system-level integration of maturation cues.

A critical next frontier is defining the temporal sequencing and relative weighting of maturation cues to recapitulate developmental trajectories. Early-stage metabolic priming may sensitize cells to subsequent mechanical loading, while premature electrical pacing in metabolically immature cells may induce maladaptive stress responses. Systematic dissection of these temporal dependencies, potentially through high-dimensional design of experiment frameworks and machine learning-guided optimization, will be essential for moving from empirical to principled maturation strategies. Across disease modeling and pharmacological applications, predictive and mechanistic power depends on adult-like myocardial fidelity. Accordingly, integrative and temporally coordinated maturation emerges as a foundational requirement, enabling the translational utility of hiPSC-CMs.

Advances in microfluidics, biomaterials, and bioprinting, coupled with genome engineering and high-resolution phenotyping, are expected to drive the development of predictive, patient-specific cardiac models. Critically, progress will depend on balancing increasing biological complexity with scalability and experimental accessibility, ensuring these platforms remain deployable for translational and high-throughput applications. Together, these efforts define a roadmap toward standardized, scalable, and biologically faithful cardiac maturation systems.

## Data Availability

No new data were created or analyzed in this study. Data sharing is not applicable to this article.
